# The effort–reward imbalance model in policing: associations with mental health and work functioning among sworn and civilian employees

**DOI:** 10.1007/s00420-026-02231-5

**Published:** 2026-08-29

**Authors:** Bjørn Lau, Knut Inge Fostervold, Brita Bjørkelo

**Affiliations:** 1 Department of Psychology, University of Oslo, Oslo, Norway; 2 Norwegian Police University College, Oslo, Norway

**Keywords:** Effort–reward imbalance, Overcommitment, Police employees, Work engagement, Job-related self-efficacy, Anxiety, Depression, Norway

## Abstract

**Objective:**

The Effort–Reward Imbalance (ERI) model is widely used to explain work-related stress and its health consequences. In policing, ERI research has primarily focused on sworn officers, while civilian employees have received limited attention. The purpose of this study was to examine whether ERI variables are associated with mental health and occupational functioning among police employees, and whether these associations differ between sworn officers and civilian personnel.

**Methods:**

This cross-sectional study included sworn police officers and civilian employees in the Norwegian police. A random sample of 4000 employees was invited to complete an online survey, and 560 employees participated. Effort, reward, and overcommitment were measured with the 16-item short ERI questionnaire, and the ERI ratio was calculated from the effort and reward scales. Anxiety symptoms, depressive symptoms, work engagement, and job-related self-efficacy were assessed using separate questionnaires. Group differences were examined using analyses of covariance, and associations between ERI predictors and outcome variables were tested using hierarchical regression analyses with interaction terms for occupational role.

**Results:**

In the covariate-adjusted analytic sample (N = 558), no significant differences were observed between sworn officers and civilian employees in ERI variables, mental health outcomes, or occupational functioning. Higher ERI ratio was associated with higher anxiety symptoms (β = 0.28) and depressive symptoms (β = 0.33) and with lower work engagement (β = − 0.27) and job-related self-efficacy (β = − 0.16). Overcommitment showed stronger associations with anxiety symptoms (β = 0.50) and depressive symptoms (β = 0.46) and was inversely associated with job-related self-efficacy (β = − 0.28) and work engagement (β = − 0.14). Occupational role did not moderate these associations.

**Conclusions:**

The findings support the ERI model by demonstrating consistent associations between effort–reward imbalance, overcommitment, mental health, and occupational functioning in policing. Neither the levels of the ERI variables nor their associations with the outcomes differed by occupational role, suggesting that ERI processes operate similarly across occupational categories within a highly regulated welfare-state context.

## Introduction

Police work involves both acute operational hazards and chronic organizational demands that can compromise employee well-being and personal accomplishment (Purba & Demou 2019; Violanti et al. 2017). Systematic reviews show that officers are frequently exposed to psychosocial stressors such as heavy workload, irregular shift work, limited organizational support, and administrative pressures (Purba & Demou 2019; Ugwu & Idemudia 2024). These exposures have been linked to psychological distress, emotional exhaustion, and physiological dysregulation—including cardiometabolic and inflammatory alterations that elevate cardiovascular risk (Franke et al. 2010; Garbarino & Magnavita 2015, 2019). At the same time, many police employees demonstrate coping and resilience strategies, deriving meaning and professional identity from their work, which may buffer the effects of chronic stress (Arble et al. 2018; Violanti et al. 2017). Thus police stress and health should be understood as a multifactorial occupational health concern, shaped by acute operational exposures, chronic organizational demands, and protective resources operating at both individual and organizational levels.

Several work-stress frameworks could be applied to police work, but they address somewhat different empirical questions. The demand–control–support model (Johnson & Hall 1988; Karasek 1979), the job demands–resources model (Bakker & Demerouti 2007; Demerouti et al. 2001), and person–environment fit theory (Edwards et al. 1998) are particularly useful for examining constellations of demands, resources, autonomy, support, and fit. By contrast, allostatic approaches (McEwen 1998) and the cognitive activation theory of stress (Ursin & Eriksen 2004) primarily address stress-response mechanisms and adaptation at a different explanatory level. The present study focuses more specifically on reciprocity in the employment relationship: whether employees’ work investment is matched by organizational and socioemotional returns, and whether such imbalance is associated with mental health and occupational functioning. For this purpose, the Effort–Reward Imbalance (ERI) model provides the most direct theoretical and measurement fit (Siegrist 1996, 2016) and allows comparison with existing police-specific ERI research reviewed below.

The ERI model proposes that strain arises when employees’ efforts are not reciprocated by adequate rewards. Importantly, high effort in itself is not assumed to be detrimental; rather, stress results from a lack of reciprocity between effort and reward. This imbalance—commonly expressed as the ERI ratio—violates the principle of reciprocity that underpins the employment relationship. Effort represents job demands such as workload and time pressure, whereas rewards encompass both material outcomes (e.g., pay, promotion prospects, job security) and socioemotional returns such as esteem, recognition, and supervisory support. The model also includes overcommitment, a personality-based tendency toward excessive work involvement and a strong need for approval, which increases vulnerability to strain. Collectively, these components provide an integrative framework for understanding contextual and dispositional sources of work-related stress.

Extensive research across occupations and national contexts supports ERI. Meta-analytic evidence shows that higher ERI ratios predict elevated risks of depression (van Vegchel et al. 2005) and cardiovascular morbidity (Eddy et al. 2017). These reviews also reveal dose–response relationships, whereby greater imbalance corresponds to proportionally higher health risks, and identify overcommitment as an amplifying factor (Eddy et al. 2017; Siegrist 2016).

Consistent with the Effort–Reward Imbalance (ERI) model, high effort–reward imbalance and overcommitment have been associated with adverse psychological outcomes in police samples. For example, Italian special police officers with high ERI ratios were almost eight times more likely to report depressive symptoms compared with those without imbalance (Garbarino et al. 2013). Among U.S. officers, both the ERI ratio and overcommitment predicted burnout symptoms of cynicism and exhaustion, whereas overcommitment alone was linked to reduced professional efficacy (Violanti et al. 2018a). Comparable associations between high ERI ratios and psychological distress have also been reported among Canadian police officers (Janzen et al. 2007).

Effort–reward imbalance (ERI) has also been related to both objective and motivational indicators of work behavior and performance. In Italian police officers, higher effort and lower reward predicted greater absenteeism, whereas higher reward and social support were associated with fewer absences (Magnavita & Garbarino 2013). Similarly, among Australian officers, low supervisory recognition—a form of low esteem reward—was linked to higher rates of absenteeism (Allisey et al. 2016). Complementary patterns are observed for motivational functioning: higher ERI ratios predicted lower work engagement, while esteem and job security rewards were associated with stronger engagement, and overcommitment was negatively related to vigor (Wolter et al. 2021).

Higher ERI ratios have been linked to greater work–family conflict (Willis et al. 2008) and poorer sleep quality, reflecting sustained psychophysiological strain in policing work (Wu et al. 2014). At the somatic level, exposure to high effort combined with low reward has been associated with elevated risk of musculoskeletal pain among German special-forces police (von dem Knesebeck et al. 2005). Longitudinal and physiological studies further suggest that ERI is related to cardiometabolic dysregulation, including metabolic syndrome and adverse metabolic profiles, highlighting the potential health consequences of prolonged effort–reward imbalance (Franke et al. 2010; Garbarino & Magnavita 2015, 2019).

Taken together, existing findings demonstrate that ERI processes have broad implications for both health and performance in policing. However, important gaps remain in the literature. Research on ERI in policing has predominantly focused on sworn and operational officers, often in high-demand or specialized units, whereas civilian personnel—such as dispatchers, analysts, and administrative specialists—have received comparatively little empirical attention despite their increasingly central organizational roles. Moreover, even when both groups are included, they are rarely analyzed separately. This gap is important because modern police organizations rely on both sworn and civilian employees to deliver core services, yet these groups may experience different combinations of work demands, recognition, status, career opportunities, and organizational support. If ERI evidence is based mainly on sworn officers, occupational health initiatives may overlook reciprocity-related stressors among civilian employees or assume that findings from operational police work apply equally across the organization. Consequently, it remains unclear whether ERI mechanisms generalize across occupational categories that differ systematically in task demands, reward structures, educational background, and organizational status within police organizations (Sørengaard et al. 2022). Clarifying this issue is important because evidence of role-specific ERI patterns would indicate a need for more targeted assessment and prevention, whereas similar patterns across groups would support broader organization-wide approaches to psychosocial risk management.

A second gap concerns the limited geographic scope of ERI research in policing. Most existing studies originate from Central and Southern Europe, North America, and East Asia, whereas Scandinavian contexts remain comparatively underrepresented (e.g., Garbarino et al. 2013; Izawa et al. 2016; Violanti et al. 2018b; Wolter et al. 2021). Moreover, available Norwegian studies have not distinguished between sworn and civilian personnel in their analyses (Sørengaard et al. 2022). This gap matters because the Norwegian police operate within a highly institutionalized and egalitarian labor-market context characterized by standardized employment conditions and strong collective regulation. Such a context may reduce variation in some ERI reward channels, such as pay and job security, while leaving other reward channels, such as esteem, recognition, support, and career opportunities, more dependent on organizational practice and occupational role. Examining ERI in this setting therefore provides a useful test of whether reciprocity-related stress processes are evident even in a relatively regulated public-sector work environment.Together, these gaps underscore the need to examine ERI processes in a Norwegian policing context and across distinct occupational roles within the same organization.

The present study addresses these empirical and contextual gaps by examining ERI processes among sworn officers and civilian employees in the Norwegian Police Service (NPS). First, we compared the two groups on core ERI variables and on a broad range of outcomes. This comparison is valuable because it can indicate whether reciprocity-related work stress should be understood primarily as a shared organizational mechanism across police employees or whether it may require more role-specific attention. Although pay and job security are comparatively standardized in the NPS, other ERI reward channels—such as esteem, recognition, career opportunities, and perceived organizational status—may vary by occupational role. Studies also indicate that different types of police work vary in prestige: both male and female police employees rate “outside” assignments, such as counter-terrorism duties, higher than “inside” work, such as investigation, and higher-prestige assignments tend to carry more status and resources, including time for training and economic benefits (Bjørkelo et al. 2022). Sworn officers’ reward structures may be shaped by police-specific education, operational experience, and professional identity, whereas civilian employees may contribute specialized administrative, analytical, technical, or legal expertise without necessarily having access to the same police-specific status and career pathways. In addition to anxiety and depressive symptoms—commonly examined ERI-related outcomes—we included work engagement and job self-efficacy, two positive indicators of occupational functioning that have received limited attention in ERI research on police employees. This approach allows for a more comprehensive assessment of ERI as a psychosocial risk factor that relates not only to strain but also to motivational resources that support sustained functioning at work.

Second, we examined whether occupational role moderates the associations between ERI and employee outcomes. Although the ERI model does not posit role-specific mechanisms, the same level of effort–reward imbalance may be experienced and interpreted differently depending on employees’ position in the organization, their professional identity, and the reward structures available to them. For example, limited recognition or unclear career prospects may have different implications for employees whose roles are anchored in police-specific professional pathways than for employees whose contributions are based on civilian specialist expertise. Conversely, if ERI associations are similar across groups, this would suggest that reciprocity-related work stress operates as a broader organizational mechanism rather than as a role-specific process. Testing moderation by occupational role therefore contributes theoretically by evaluating the generalizability of ERI mechanisms across heterogeneous roles within the same organization, and practically by informing whether preventive efforts should target shared organizational conditions, role-specific reward structures, or both.

## Aims and hypotheses

The study had two research questions and one theory-based hypothesis. RQ1 and RQ2 concerned group differences and moderation by occupational role. Because plausible arguments could be made for both similarity and difference between sworn officers and civilian employees, and because prior research provided limited grounds for directional role-based predictions, these issues were formulated as exploratory research questions rather than hypotheses. H1 was based on assumptions from the ERI model and concerned the expected overall associations between ERI predictors and mental health and occupational functioning. Across these aims, ERI thus occupies two conceptual positions: the ERI variables serve as outcomes of occupational role in RQ1, whereas the ERI predictors serve as exposures in relation to the outcome variables in RQ2 and H1, reflecting two levels of explanation.

### RQ1.

Do sworn and civilian police employees differ in ERI variables (Effort, Reward subscales, ERI ratio, Overcommitment), mental health outcomes (anxiety and depression), or occupational functioning (work engagement and job self-efficacy)?

### RQ2.

Does occupational role (sworn vs. civilian) moderate the associations between ERI predictors (ERI ratio, Overcommitment) and outcome variables?

### H1.

Based on the ERI model, higher ERI ratios and higher Overcommitment were expected to be associated with poorer mental health (higher anxiety and depression) and lower occupational functioning (reduced work engagement and job-related self-efficacy) across the full sample.

## Methods

### Participants and procedure

The study was conducted in the NPS, which employs approximately 17,100 staff across 12 police districts and several specialized units. In August 2021, a simple random sample of 4000 employees was drawn from the NPS employee registry, with permission from the National Police Directorate (reference #201900307), and invited to participate in an online survey. Each selected employee received an email containing a secure survey link and detailed study information, followed by an electronic informed consent form. Employees could proceed to the questionnaire only after providing consent. The survey was distributed on 23 August 2021 and remained open for approximately three weeks, with two email reminders (26 August and 6 September 2021) sent to nonrespondents to maximize participation. The survey was conducted as part of the Norwegian Police Study, a broader research project on working conditions, stress, and health in the NPS (Rabbing et al. 2025). Because the survey was designed to cover a wide range of working conditions and health outcomes across the entire organization, the present study draws on the modules assessing effort–reward imbalance, mental health, work engagement, and job-related self-efficacy.

A total of 560 employees completed the survey, corresponding to a response rate of 14%. The respondent sample included 346 sworn police officers (61.8%) and 214 civilian employees (38.2%). Participants were drawn from across the national police organization, including both police districts and specialized units. In the full respondent sample, 408 participants (72.9%) worked in police districts and 152 (27.1%) in specialized units; 482 participants (86.1%) reported an urban work location and 78 (13.9%) a rural work location. The sample also included variation in educational background, including employees with police academy education and employees with other educational backgrounds.

Available aggregate registry-based comparisons of the same survey sample have been reported previously as part of the broader Norwegian Police Study (Rabbing et al. 2025, Table 1). These comparisons showed that the respondent sample was broadly comparable to both the invited sample and the total NPS workforce with respect to gender and occupational role. Women comprised 43.2% of respondents, compared with 45.4% of the invited sample and 46.2% of the total workforce. Sworn officers comprised 61.8% of respondents, compared with 60.0% of the invited sample and 59.8% of the total workforce. Younger employees were somewhat underrepresented among respondents, with employees below 30 years comprising 7.1% of respondents compared with 12.1% of the invited sample and 12.3% of the total workforce. Employees in specialized units were overrepresented among respondents, comprising 27.1% of respondents compared with 17.7% of the invited sample and 17.8% of the total workforce.

**Table 1:** Group differences between sworn police officers (SPOs) and civilian employees (Civilians) in sociodemographic variables (N = 560)

Variable	Civilians(n = 214)	SPOs(n = 346)	Test	*p*	Effect size(Cohen’s d/Cramér’s V)
Age (years)	47.8 (9.2)	43.1 (10.1)	*t*(558) = 5.40	< 0.001	*d* = 0.47
Length of service (years)	11.1 (9.1)	17.1 (10.8)	*t*(558) = − 6.70	<0.001	*d* = − 0.58
Female (%)	63.6	30.8	*χ*^*2*^(1) = 57.57	<0.001	V = 0.32
Married/cohabiting (%)	76.6	84.1	*χ*^*2*^(1) = 4.84	0.028	V = 0.09

Values are mean (standard deviation) unless otherwise noted. Positive Cohen’s d values indicate higher means for civilians; negative Cohen’s d values indicate higher means for sworn officers. Cramér’s V is reported as the measure of association for categorical variables. Conventional cut-off points for Cramér’s V were interpreted as 0.10 = small, 0.30 = medium, and 0.50 = large

### Measures

#### Demographic and occupational variables

Participants provided demographic information on gender, age, marital or cohabitation status, education level, geographic work location, and organizational unit. Gender was self-reported as female, male, or other. Fewer than five participants reported the category other and were therefore excluded from analyses in which gender was included as a covariate, resulting in an analytic sample of 558. Marital status was dichotomized into cohabiting with a partner, including married, cohabiting, or registered partner, versus not cohabiting, including single, separated or divorced, and widowed.

Occupational role was assessed by asking participants whether they were employed in a sworn police position or a civilian position. In the Norwegian context, this distinction largely reflects whether employees have completed the bachelor-level police education delivered by the Norwegian Police University College, which includes operational field training. Sworn officers should therefore be understood as police-educated employees working across police-professional roles, including frontline policing, emergency response, investigation, crime prevention, and related operational or investigative duties. Civilian employees should be understood as employees in non-sworn positions, including administrative, analytical, technical, forensic, civil-enforcement, governance-related, and other specialist support functions. Because police education and occupational role overlap closely within the Norwegian police, the sworn/civilian distinction was used as the primary grouping variable in the analyses. The survey did not include sufficiently detailed job-title categories to distinguish specific occupational subgroups, such as patrol officers, detectives, lawyers, or communication staff, in the statistical models. The role variable should therefore be interpreted as a broad institutional role classification rather than as a fine-grained job-task taxonomy.

#### Background characteristics and covariate selection

Background characteristics by occupational role are presented in Table 1. Sworn officers and civilian employees differed on several background variables. Civilian employees were older, had shorter length of service, and included a higher proportion of women, whereas sworn officers were more likely to be married or cohabiting. Effect sizes were small to moderate for age (d = 0.47) and length of service (d = − 0.58), medium for gender (Cramér’s V = 0.32), and below the conventional cut-off for a small effect for marital/cohabitation status (Cramér’s V = 0.09). Because these variables differed between occupational groups and could be associated with the study outcomes, age, gender, marital status, and length of service were included as covariates in the adjusted group comparisons and regression analyses.

#### Effort–reward imbalance and overcommitment

Work-related stress was assessed with the short version of the Effort–Reward Imbalance (ERI) questionnaire (Siegrist et al. 2009). The instrument consists of three items measuring perceived effort and seven items measuring perceived reward across the three domains of esteem (two items), job security (two items), and job promotion opportunities (three items; Siegrist et al. 2004). In addition, the questionnaire includes a six-item Overcommitment subscale that reflects a compulsive, work-related over-involvement (Siegrist 1996). All items were rated on a 4-point Likert scale from 1 (“strongly disagree”) to 4 (“strongly agree”).

Mean scores were calculated for Effort, each of the three Reward subdimensions, total Reward, and Overcommitment, with higher scores indicating higher perceived effort, higher perceived reward, and greater overcommitment. An effort–reward imbalance ratio was also calculated to quantify the mismatch between effort and reward (Siegrist et al. 2004). The ratio was computed as the Effort score divided by the Reward score, such that values greater than 1.0 denote a problematic imbalance (i.e., high effort relative to low reward). The ERI scales have demonstrated good psychometric properties in diverse worker populations (Siegrist et al. 2009). In the present sample, internal consistency was satisfactory (Cronbach’s α = 0.74 for Effort, 0.79 for Reward, and 0.85 for Overcommitment).

#### Job-related self-efficacy

Perceived ability to handle work demands was assessed with a context-specific adaptation of the General Self-Efficacy Scale (GSE; Schwarzer 1992; Schwarzer & Jerusalem 1995). The standard 10 GSE items were retained but rephrased to reflect the policing work context, based on a Norwegian translation by Røysamb (1997). Participants rated each item on a 4-point scale ranging from 1 (“not at all true”) to 4 (“exactly true”). Responses were summed to create an overall job-related self-efficacy score (range 10–40), with higher scores indicating greater confidence in the ability to cope with work-related challenges. The scale demonstrated good internal consistency in the present sample (Cronbach’s α = 0.85).

#### Work engagement

Work engagement was measured with the Utrecht Work Engagement Scale–9 (UWES-9; Schaufeli et al. 2006). The UWES-9 consists of nine items covering three subdimensions of engagement—vigor, dedication, and absorption—with three items each. In the present study, we used the overall engagement score. Participants rated how often they experienced each engagement indicator on a 7-point scale ranging from 0 (“never”) to 6 (“always”). Scores were calculated as the mean of all nine items, with higher scores indicating greater work engagement. The UWES-9 is a widely used and well-validated measure in occupational health research (Schaufeli et al. 2006). In the current sample, the scale demonstrated excellent internal consistency (Cronbach’s α = 0.95).

#### Anxiety symptoms

Symptoms of anxiety were measured with the Generalized Anxiety Disorder Scale–7 (GAD-7; Spitzer et al. 2006). The GAD-7 consists of seven items asking how often in the past two weeks respondents had been bothered by core anxiety symptoms (e.g., feeling nervous or “on edge,” not being able to control worrying, trouble relaxing). Items were rated on a 4-point scale from 0 (“not at all”) to 3 (“nearly every day”) and summed to produce a total score ranging from 0 to 21, with higher scores indicating greater symptom severity. The scale demonstrated excellent consistency in the present sample (Cronbach’s α = 0.90).

#### Depressive symptoms

Depressive symptoms were assessed with the Patient Health Questionnaire–9 (PHQ-9; Kroenke et al. 2001), a widely used measure of depression in population research. Participants reported how often over the past two weeks they had experienced each of nine DSM-defined depression symptoms (e.g., little interest or pleasure in activities, feeling down or hopeless, trouble sleeping, low energy, poor appetite or overeating, thoughts of self-harm). Items were rated on a 4-point scale from 0 (“not at all”) to 3 (“nearly every day”), and summed to produce a total score ranging from 0 to 27, with higher scores indicating greater severity of depressive symptoms. The PHQ-9 has been validated, including in Norwegian samples (Wisting et al. 2021), and demonstrated high internal consistency in the present study (Cronbach’s α = 0.85).

## Statistical analyses

Data analyses were designed to address the study’s research questions and hypothesis in two main steps: (a) testing group differences in ERI variables and outcome variables (RQ1), and (b) evaluating potential moderation by occupational role and associations between ERI predictors and mental health and occupational outcomes (RQ2; H1). Background characteristics were examined descriptively to characterize the sample and to determine covariates for the adjusted analyses.

All statistical analyses were conducted using IBM SPSS Statistics (Version 29). Descriptive statistics were examined for all study variables. Item-level missing data were negligible, as the online questionnaire prompted responses to all relevant items; analyses were therefore based on complete cases. Assumptions of normality and homogeneity of variance were examined using Q–Q plots and Levene’s tests. The results indicated that the data met these assumptions sufficiently for the planned analyses.

### Descriptive and preliminary analyses

Descriptive statistics and preliminary analyses were conducted to characterize the sample and examine whether sworn officers and civilian employees differed on key background variables. Independent-samples t-tests were used for continuous variables (age and length of service), and chi-square tests were used for categorical variables (gender and marital status). Age, gender, marital status, and length of service were included as covariates in subsequent analyses as potentially relevant background factors that could confound associations between occupational role and the study outcomes.

### Group comparisons (RQ1)

To address the first research question, sworn officers and civilian employees were compared on (a) the ERI variables—Effort, the three Reward subdimensions (esteem, job security, and job promotion), Overcommitment, and the ERI ratio (Effort ÷ Reward)—and (b) the outcome variables—anxiety, depression, work engagement, and job-related self-efficacy.

For each variable, a one-way analysis of covariance (ANCOVA; UNIANOVA procedure in SPSS) was conducted, controlling for gender, age, marital status, and tenure. Adjusted means and pairwise comparisons were obtained from estimated marginal means using least significant difference (LSD) adjustment. Homogeneity of variance was evaluated using Levene’s test. When Levene’s test indicated unequal variances, the corresponding group comparison was re-examined with a Welch-adjusted *F* test (ONEWAY procedure with the WELCH option) to ensure robustness. Partial *η*2 values were reported as effect-size estimates for all adjusted mean differences.

### Associations between ERI predictors and outcomes (RQ2; H1)

To examine associations between ERI predictors and outcome variables, hierarchical multiple regression analyses were conducted. Potential moderation by occupational role was also tested. Separate models were specified for the overall ERI ratio and for Overcommitment, yielding eight models across the four outcome variables. This approach was chosen because ERI ratio and Overcommitment represent conceptually distinct core predictors within the ERI model, and because the aim was to examine whether each predictor was associated with the outcome variables and whether these associations differed between sworn officers and civilian employees. To enhance comparability with prior ERI research and to keep the models parsimonious, the association analyses focused on ERI ratio and Overcommitment as the two central ERI indicators.

In each model, demographic covariates (gender, age, marital status, and tenure) were entered at Step 1. Occupational role (0 = civilian, 1 = sworn officer) was entered at Step 2, and the grand-mean–centered ERI ratio or Overcommitment predictor was entered at Step 3. The interaction term between the centered ERI variable and occupational role was entered at Step 4. Results reported refer to the final step (Step 4) of the hierarchical regression, which included the interaction term.

All continuous variables were grand-mean centered so that each participant’s score represented a deviation from the overall sample mean (e.g., *Overcommitment*_C_ = *Overcommitment* – *Overcommitment*_M_). This procedure reduces multicollinearity between main effects and interaction terms and facilitates interpretation of regression coefficients.

If a significant interaction term was detected at Step 4, follow-up simple-slope analyses were conducted within each occupational role to probe associations between the ERI predictor and outcome variables. Model assumptions of linearity, normality, homoscedasticity, and multicollinearity were examined. All assumptions were satisfied, with variance-inflation factors below 2 and tolerance values above 0.50. Model explanatory power was evaluated using the total *R*^2^ from the final step (Step 4) of each hierarchical regression.

### Reporting and effect size

All hypothesis tests were two-tailed (*α* = 0.05). Descriptive comparisons between sworn officers and civilian employees (Table 1) were based on *t*-tests and *χ*^2^ tests of crude means and proportions. For these analyses, Cohen’s d was reported for continuous variables (age and length of service) and Cramér’s V for categorical variables (gender and marital status).

In group comparisons adjusted for covariates (Table 2), analyses of covariance (ANCOVAs) were used to compare adjusted mean scores on ERI variables and outcome variables, with associated *p*-values and partial *η*^2^ effect sizes reported.

**Table 2:** Adjusted group differences in ERI variables and outcome variables (ANCOVA results)

Variable	Adj. M (SE)Civilian	Adj. M (SE)Sworn	*F*(1, 552)	*p*	Partial η^2^	Levene’s *F*	Welch *F*(df)
**ERI model variables**							
Effort	2.63 (0.05)	2.73 (0.04)	1.83	0.177	0.003	6.20*	2.41 (≈ 349)
Reward – Esteem	2.87 (0.06)	2.89 (0.04)	0.07	0.787	0.000	0.13	–
Reward – Job Security	2.93 (0.06)	2.92 (0.04)	0.01	0.945	0.000	7.38*	0.01 (≈ 326)
Reward – Promotion	2.27 (0.06)	2.27 (0.04)	0.00	0.969	0.000	1.61	–
Overcommitment	2.01 (0.05)	2.08 (0.04)	1.02	0.312	0.002	3.68	–
ERI ratio (Effort ÷ Reward)	1.09 (0.04)	1.10 (0.03)	0.04	0.845	0.000	2.78	–
**Health and work outcomes**							
Anxiety (GAD-7)	3.81 (0.30)	3.37 (0.22)	1.11	0.294	0.002	13.07*	4.08 (≈ 338)
Depression (PHQ-9)	4.37 (0.35)	3.61 (0.26)	2.47	0.117	0.004	26.58*	7.01 (≈ 327)
Work engagement (UWES)	4.11 (0.11)	3.94 (0.09)	1.19	0.275	0.002	7.61*	0.66 (≈ 387)
Job self-efficacy (GPSE)	32.00 (0.33)	32.87 (0.25)	3.58	0.059	0.006	0.84	–

Adj. M = covariate-adjusted mean; SE = standard error. Higher scores reflect higher observed values on the respective scales. Levene’s test was used to assess variance homogeneity; asterisks (*) denote significant Levene’s tests (*p* < 0.05). Welch-adjusted *F* values are reported where Levene’s test was significant to ensure robustness of group comparisons. Partial η^2^ values of 0.01, 0.06, and 0.14 were interpreted as small, medium, and large effect sizes, respectively

For regression analyses (Table 3), unstandardized coefficients (B) with standard errors and standardized coefficients (β) from the final model (Step 4) were presented, along with model R2 statistics. Statistical significance is indicated in the table.

**Table 3:** Hierarchical regression analyses predicting anxiety (GAD-7), depression (PHQ-9), work engagement (UWES), and job self-efficacy (GPSE) (N = 558)

Predictor	Anxiety (GAD-7)	Depression (PHQ-9)	Job-related self-efficacy (GPSE)	Work engagement (UWES)
**ERI-ratio model (Step 4)**				
Gender	− 0.93 (0.32) [− 0.12]*	− 0.58 (0.37) [− 0.07]	0.92 (0.36) [0.11]*	0.14 (0.12) [0.05]
Age (centered)	− 0.03 (0.03) [− 0.07]	− 0.01 (0.03) [− 0.03]	0.01 (0.03) [0.01]	0.01 (0.01) [0.04]
Married/cohabiting	− 0.37 (0.39) [− 0.04]	− 1.22 (0.45) [− 0.11]*	0.52 (0.44) [0.05]	0.24 (0.15) [0.07]
Length of service (centered)	− 0.02 (0.02) [− 0.05]	− 0.03 (0.03) [− 0.07]	− 0.03 (0.03) [− 0.07]	0.01 (0.01) [0.08]
Occupational role	− 0.45 (0.41) [− 0.06]	− 0.75 (0.47) [− 0.09]	0.86 (0.46) [0.10]	− 0.16 (0.15) [− 0.06]
ERI-ratio (centered)	**2.23 (0.48) [0.28]**	**3.00 (0.56) [0.33]**	− 1.38 (0.55) [− 0.16]*	**− 0.80 (0.18) [− 0.27]**
ERI × Group	− 0.37 (0.64) [− 0.04]	− 1.15 (0.74) [− 0.10]	0.85 (0.73) [0.07]	− 0.05 (0.24) [− 0.01]
*Model R*2 (Step 4)	0.10	0.11	0.05	0.10
**Overcommitment model (Step 4)**				
Gender	− 0.71 (0.29) [− 0.10]*	− 0.37 (0.35) [− 0.04]	0.78 (0.36) [0.10]*	0.14 (0.13) [0.05]
Age (centered)	− 0.04 (0.02) [− 0.12]	− 0.03 (0.03) [− 0.07]	0.02 (0.03) [0.04]	0.01 (0.01) [0.06]
Married/cohabiting	− 0.44 (0.36) [− 0.05]	− 1.27 (0.43) [− 0.12]*	0.56 (0.43) [0.05]	0.22 (0.15) [0.06]
Length of service (centered)	− 0.01 (0.02) [− 0.04]	− 0.02 (0.03) [− 0.06]	− 0.03 (0.03) [− 0.08]	0.01 (0.01) [0.07]
Occupational role	− 0.63 (0.37) [− 0.08]	− 0.93 (0.44) [− 0.11]*	0.98 (0.45) [0.12]*	− 0.16 (0.16) [− 0.06]
Overcommitment (centered)	**3.02 (0.34) [.50]**	**3.17 (0.41) [0.46]**	**− 1.84 (0.41) [− 0.28]**	− 0.32 (0.15) [− 0.14]*
Overcommitment × Group	− 0.43 (0.45) [− 0.05]	− 0.78 (0.54) [− 0.09]	0.47 (0.55) [0.05]	0.17 (0.19) [0.06]
*Model R*2 (Step 4)	0.25	0.19	0.09	0.03

Unstandardized coefficients (*B*) with standard errors in parentheses and standardized coefficients (*β*) in brackets, all from the final model (Step 4). Covariates (gender, age, marital status, length of service) entered at Step 1; occupational role at Step 2; ERI predictor (ERI-ratio or Overcommitment) at Step 3; interaction term at Step 4. Predictors were grand-mean–centered. Gender coded 1 = female, 2 = male; Married/cohabiting coded 0 = not cohabiting (single/divorced/widowed), 1 = married or cohabiting; Occupational role coded 0 = civilian, 1 = sworn officer. Boldface indicates *p* < 0.001; * indicates *p* < 0.05. All tests were two-tailed

## Results

### Group differences in ERI variables and outcome variables (RQ1)

To address RQ1, analyses of covariance (ANCOVAs) were conducted to examine potential role differences between sworn officers and civilian employees across ERI variables and outcome variables, controlling for gender, age, marital status, and length of service (Table 2).

No significant group differences were observed on any ERI variable (Effort, the three Reward subscales, Overcommitment, or the overall ERI ratio) or outcome variable (anxiety, depression, work engagement, and job-related self-efficacy). Adjusted mean scores were nearly identical across groups (all *p* ≥ 0.06, partial *η*^2^ ≤ 0.006). Although the difference in job self-efficacy approached significance (*p* = 0.059), this trend did not reach conventional levels of statistical significance.

When heterogeneity of variance was detected, Welch-adjusted tests confirmed the same nonsignificant pattern. These results suggest that sworn and civilian police personnel experience comparable levels of effort–reward imbalance, overcommitment, anxiety and depressive symptoms, work engagement, and job-related self-efficacy.

### Associations between ERI predictors and outcome variables (RQ2; H1)

Hierarchical multiple regressions were conducted to test whether occupational role moderated the associations between ERI predictors and outcome variables (RQ2) and to examine the hypothesized associations between ERI predictors and outcome variables (H1; see Table 3).

Consistent with the ERI model (Siegrist 1996, 2016), higher ERI imbalance was associated with poorer mental health and reduced occupational functioning. Specifically, for the ERI-ratio models, greater imbalance predicted higher anxiety (*B*= 2.23, SE = 0.48, *β* = 0.28, *p* < 0.001) and depression (*B* = 3.00, SE = 0.56, *β* = 0.33, *p* < 0.001), and lower work engagement (*B* = − 0.80, SE = 0.18, *β* = − 0.27, *p* < 0.001) and job self-efficacy (*B* = − 1.38, SE = 0.55, *β* = − 0.16, *p* = 0.013). The ERI-ratio models accounted for approximately 5–11% of the variance in the respective outcomes.

Similarly, for the Overcommitment models, higher Overcommitment predicted greater anxiety (*B* = 3.02, SE = 0.34, *β* = 0.50, *p* < 0.001) and depression (*B* = 3.17, SE = 0.41, *β* = 0.46, *p* < 0.001), as well as lower work engagement (*B* = − 0.32, SE = 0.15, *β* = − 0.14, *p* = 0.027) and job self-efficacy (*B* = − 1.84, SE = 0.41, *β* = − 0.28, *p* < 0.001). These models explained between 3 and 25% of the variance in employee outcomes.

Across all models, the ERI × role and Overcommitment × role interaction terms were nonsignificant (all *p* ≥ 0.12), indicating that the associations between ERI predictors and mental health and occupational functioning outcomes did not differ between sworn officers and civilian personnel. Thus, RQ2 yielded no support of moderation by occupational role, whereas H1 was supported.

## Discussion

The present study applied the ERI framework to a diverse sample of Norwegian police employees with a twofold aim: to examine whether sworn and civilian employees differ in their perceived levels of effort, reward, and overcommitment, and whether these ERI variables were associated with mental health and occupational functioning across the two groups. Overall, the findings supported the ERI model (Siegrist 1996, 2016): higher imbalance and higher overcommitment were consistently associated with greater anxiety and depressive symptoms as well as lower work engagement and job-related self-efficacy. Importantly, after adjustment for potential confounders, occupational role did not moderate these associations, suggesting that the psychosocial processes captured by the ERI model operate similarly across distinct job categories within the same organizational context.

context.

Conceptually, ERI occupied two distinct positions in the present design, reflecting two levels of explanation. In the group comparisons (RQ1), the ERI variables were treated as outcomes of employees’ structural position, examining whether occupational role shapes the level of experienced effort, reward, and overcommitment. In the association analyses (RQ2, H1), the ERI predictors served as the proximal psychosocial exposure theorized by the model to affect mental health and occupational functioning (Siegrist 1996, 2016). This two-step structure mirrors a core logic of occupational health epidemiology, in which distal organizational conditions shape proximal psychosocial exposures, which in turn relate to health. In the following, we therefore discuss ERI as an outcome when addressing why levels did not differ across occupational roles, and as an exposure when interpreting its associations with anxiety, depression, work engagement, and self-efficacy. Given the cross-sectional design, the latter directionality is model-derived rather than established by the data, a point we return to when discussing limitations.

Consistent with core predictions of the ERI model, both effort–reward imbalance and overcommitment were associated with elevated anxiety and depressive symptoms. These findings align with meta-analytic and physiological evidence linking ERI to depressive disorders, dysregulated stress responses, and inflammatory processes (Eddy et al. 2017; Izawa et al. 2016; van Vegchel et al. 2005). The particularly strong associations observed for overcommitment replicate findings from prior police studies in Italy and the United States (Garbarino et al. 2013; Violanti et al. 2018a) and support Siegrist’s conceptualization of overcommitment as an intrinsic vulnerability that amplifies strain (Siegrist 1996). In contrast, the associations of the ERI ratio were somewhat smaller but extended across both health-related and work-related outcomes, suggesting that imbalance is related not only to psychological well-being but also to employees’ capacity to function effectively at work. Taken together, these patterns are consistent with a central premise of the ERI model: it is the perceived lack of reciprocity between effort and reward, rather than high effort alone, that constitutes the critical psychosocial stressor.

By including work engagement and job-related self-efficacy, this study extends ERI research in policing beyond its traditional focus on negative health outcomes. The inverse associations between ERI and work engagement are consistent with prior studies showing that esteem-related rewards and job security are central for sustaining vitality and dedication among police employees (Wolter et al. 2021; Allisey et al. 2016). Similarly, the association between imbalance and lower self-efficacy aligns with social-cognitive perspectives emphasizing the role of recognition and feedback in reinforcing beliefs about competence and control (Bandura 1997). In policing, where on-the-job feedback (Dahl et al. 2023; Dahl & Damen 2026) and public feedback may vary, insufficient reward may undermine both intrinsic motivation and confidence in one’s ability to meet work demands. These findings therefore position ERI not only as a model of stress and ill-health, but also as a framework for understanding motivational erosion and reduced occupational functioning in demanding work systems.

A central contribution of the present study is twofold. First, sworn and civilian employees reported similar levels of effort, reward, and overcommitment—the ERI variables viewed as outcomes of occupational position (RQ1). Second, the associations between the ERI predictors and the outcome variables were of similar magnitude in both groups—ERI viewed as a psychosocial exposure (RQ2). Rather than challenging ERI theory, this pattern invites contextual interpretation. Viewed from the antecedent side—ERI as an outcome of organizational conditions—features of Norwegian policing such as standardized employment conditions, strong collective regulation, and the egalitarian labor-market institutions characteristic of the Nordic countries (Hellum et al. 2024; Hvid & Falkum 2019) may reduce systematic disparities in both demands and rewards across occupational boundaries. This interpretation is consistent with recent Norwegian findings showing that sworn and civilian personnel report largely similar exposure to organizational and operational stressors (Rabbing et al. 2025). The absence of moderation, by contrast, concerns the exposure side of the model: it indicates that where imbalance does occur, its associations with health and functioning are of similar magnitude in both groups, speaking to the generality of the mechanism rather than to the structural equalization of exposure. Taken together, these findings suggest that shared organizational structures equalize the level of effort–reward imbalance across occupational roles, while the ERI model explains why the imbalance that remains is nonetheless consequential for health and functioning.

Several methodological features strengthen confidence in these conclusions. Predictors were centered to reduce multicollinearity, and analytic assumptions were carefully examined and met. The sample size provided adequate power to detect small-to-moderate interaction effects, and results were robust across analytic specifications. Nevertheless, limitations warrant consideration.

The cross-sectional design precludes causal inference in either direction. In particular, reverse causality cannot be ruled out: employees experiencing anxiety or depressive symptoms may perceive and report less reciprocity in everyday work exchanges, rendering self-reported ERI partly an outcome of affective state rather than solely an exposure. Prospective evidence nonetheless supports the direction from effort–reward imbalance to subsequent ill-health as the dominant pathway (Rugulies et al. 2017), suggesting that reverse causation is unlikely to fully account for the observed associations. In addition, reliance on self-report measures may inflate associations through common method variance (Purba & Demou 2019).

The response rate of 14% also warrants a more detailed assessment of representativeness than a general acknowledgment of selection bias. As described in the Methods section, aggregate registry-based comparisons indicate that respondents were broadly comparable to both the invited sample and the total NPS workforce with respect to gender and the proportion of sworn versus civilian employees, whereas younger employees were somewhat underrepresented and employees in specialized units overrepresented (Rabbing et al. 2025). The similar sworn/civilian composition among respondents, invitees, and the workforce suggests that differential participation by occupational role is unlikely to explain the absence of group differences. Nevertheless, self-selection on unmeasured characteristics remains possible within both groups. If employees with better mental health and higher work engagement were more likely to participate, restricted variance in both the ERI variables and the outcome variables would be expected, which would tend to attenuate rather than inflate the observed associations. Moreover, low response rates do not translate into nonresponse bias in any straightforward manner (Groves & Peytcheva 2008). Even so, the absolute levels of symptoms and engagement reported here should be generalized to the broader police workforce with caution.

In addition, the broad categorization of employees as “sworn” or “civilian” merits closer consideration. As described in the Methods section, the survey was conducted as part of the broader Norwegian Police Study and was designed to cover working conditions and health across the entire organization; combined with the need to protect respondent anonymity in a national police sample, this precluded more fine-grained job-title measures. The binary classification captures the institutionally primary distinction in Norwegian policing—police education and its associated role and career structures—but likely masks meaningful heterogeneity within each group, for example between patrol officers, detectives, and leaders among sworn personnel, and between legal, analytical, and administrative specialists among civilian employees. If subgroups within each category experience effort–reward conditions differently, such heterogeneity would tend to obscure subgroup-specific patterns and attenuate role differences at the aggregate level. Future research should therefore employ finer-grained role classifications, alongside longitudinal designs incorporating objective indicators such as sickness absence or physiological measures. Such research should also examine the content of the imbalance: even where overall levels are comparable, the specific efforts invested and the rewards at stake—for example operational risk and public visibility versus caseload and specialist recognition—may differ between and within the two groups, with correspondingly different implications for preventive action.

Finally, the timing of the data collection warrants comment. The survey wasadministered in late August and early September 2021, during the late phase of the COVID-19 pandemic in Norway and in the weeks before the remaining national restrictions were lifted on 25 September 2021. Pandemic conditions may have influenced responses in several ways. Studies of police employees during the pandemic have documented elevated stress and additional demands among European officers (Frenkel et al. 2021), and parts of the Norwegian police workforce carried pandemic-related tasks such as border control and enforcement of infection-control regulations, which may have elevated perceived effort. At the same time, several civilian functions were performed partly from home—a shift shown to affect Norwegian police employees’ social contact with colleagues (Langvik et al. 2021)—potentially altering perceptions of demands, support, and recognition. Pandemic-related strain and restricted social contact may also have raised levels of anxiety and depressive symptoms relative to non-pandemic conditions. Because the present study concerns associations between the ERI predictors and the outcome variables rather than prevalence estimates, the main conclusions are less vulnerable to such general level shifts. Nevertheless, the pandemic context may have operated as a shared background factor influencing both work perceptions and symptom reporting, and the absolute levels reported here may not generalize to non-pandemic periods.

Despite these limitations, the study has several notable strengths. It is among the few studies to examine ERI simultaneously in sworn and civilian police employees within the same national organization, and to do so using validated measures of both mental health and occupational functioning. As a Scandinavian contribution, the study extends ERI research to a welfare-state context characterized by high job security and institutional fairness (Hellum et al. 2024; Hvid & Falkum 2019). That the associations of imbalance with health and functioning persisted under such conditions underscores the general relevance of the model: while structural context may shape how much imbalance employees experience, the correlates of experienced imbalance appear consistent across contexts—suggesting a fundamental psychosocial mechanism rather than a feature of precarious or weakly regulated work environments alone. At the same time, the modest effect sizes leave open the possibility—testable only in comparative designs across labor-market contexts—that equitable organizational structures limit employees’ exposure to imbalance, without eliminating its psychological correlates where imbalance occurs.

From an occupational health perspective, the findings highlight ERI as a useful framework for preventive action in policing. Evidence from other police settings indicates that low rewards—particularly limited recognition and social support—are associated with higher absenteeism and lower work engagement (Magnavita & Garbarino 2013; Allisey et al. 2016; Wolter et al. 2021), suggesting that reciprocity-oriented interventions may have implications for both health and work functioning. Such interventions operate on the antecedent side of the ERI model—modifying the conditions under which imbalance arises—whereas their intended benefits lie on the consequence side, in employee health and functioning. In public-sector organizations where some rewards, such as pay or job security, may be relatively fixed, feasible levers include strengthening fair and transparent procedures, ensuring timely supervisor feedback and recognition, and supporting predictable competence and career-development pathways. Because ERI associations were comparable across sworn and civilian employees, such initiatives are likely relevant across the workforce and can be integrated into routine psychosocial risk management and organizational development in policing.

## Conclusion

In sum, this study shows that effort–reward imbalance and overcommitment are consistently associated with anxiety, depressive symptoms, work engagement, and job-related self-efficacy among Norwegian police employees. The absence of role differences—in both the level of effort–reward imbalance and the strength of its associations with the outcome variables—supports the interpretation that ERI operates as a shared psychosocial mechanism across diverse roles, with similar exposure levels plausibly reflecting the standardized employment conditions of Norwegian policing. By integrating mental health and motivational outcomes, the findings contribute to a more comprehensive understanding of psychosocial risk in contemporary policing and underscore the relevance of reciprocity and fairness as central components of occupational health.

## Data Availability

The data are not publicly available due to the sensitive nature of the research and because participants did not consent to public sharing of their data.
